# Resolving Affinity Purified Protein Complexes by Blue Native PAGE and Protein Correlation Profiling

**DOI:** 10.3791/55498

**Published:** 2017-04-01

**Authors:** Mercedes Pardo, Daniel Bode, Lu Yu, Jyoti S. Choudhary

**Affiliations:** ^1^Proteomic Mass Spectrometry, Wellcome Trust Sanger Institute

**Keywords:** Biochemistry, Issue 122, Blue native PAGE, protein correlation profiling, multiprotein complex, affinity purification, quantitative mass spectrometry, interactome, AP-MS, protein interaction

## Abstract

Most proteins act in association with others; hence, it is crucial to characterize these functional units in order to fully understand biological processes. Affinity purification coupled to mass spectrometry (AP-MS) has become the method of choice for identifying protein-protein interactions. However, conventional AP-MS studies provide information on protein interactions, but the organizational information is lost. To address this issue, we developed a strategy to unravel the distinct functional assemblies a protein might be involved in, by resolving affinity-purified protein complexes prior to their characterization by mass spectrometry. Protein complexes isolated through affinity purification of a bait protein using an epitope tag and competitive elution are separated through blue native electrophoresis. Comparison of protein migration profiles through correlation profiling using quantitative mass spectrometry allows assignment of interacting proteins to distinct molecular entities. This method is able to resolve protein complexes of close molecular weights that might not be resolved by traditional chromatographic techniques such as gel filtration. With little more work than conventional AP-geLC-MS/MS, we demonstrate this strategy may in many cases be adequate for obtaining protein complex topological information concomitantly to identifying protein interactions.

**Figure:**
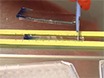


## Introduction

In cells, most proteins perform their functions through transitory protein-protein interactions or by forming stable protein assemblies. Characterizing protein interactions is crucial for fully understanding cellular processes. Affinity purification in combination with mass spectrometry (AP-MS) is one of the most commonly applied strategies to identify native protein interactions. Significant improvements in instrument capabilities achieved in the last decade have made this approach extremely powerful. It is important to note that the interactions identified by AP-MS experiments include a mixture of direct and indirect associations between bait and preys. In addition, often proteins take part in several different complexes within the same cellular context, which might have different biological roles, and therefore the interactors that are identified by AP-MS might represent a mix of distinct protein assemblies or functional entities. It is not possible to derive such topological information *a priori* from the mono-dimensional lists of proteins generated by simple AP-MS experiments. However, the technique can be exploited further to define the architecture of protein complexes by combining it with one or more methods to resolve these assemblies.

In order to resolve the topology of protein interactions identified through AP-MS, several strategies have been applied. One approach is to perform iterative AP-MS experiments using the preys identified in a previous round of experiments as baits 1. Although very informative, this is quite a labor intensive task both experimentally and analytically. Protein cross-linking in combination with mass spectrometry is increasingly being used to derive topological information on protein complexes 2,3,4,5. However, computational analysis of cross-linked peptides still remains a challenging task and hence is the bottleneck in the workflow. The advent of MS-cleavable cross-linking reagents should facilitate mapping of the amino acid residues that are in close proximity in interacting proteins 6,7. Another alternative is to combine affinity purification with prior orthogonal separation techniques 8,9. Chromatographic fractionation by gel filtration or ion exchange, or sucrose gradient fractionation have also recently been used in combination with quantitative mass spectrometry to describe multiprotein complexes at a system-wide level, by-passing the complex isolation step 10,11,12,13. Blue native polyacrylamide gel electrophoresis (BN-PAGE) has been widely applied to investigate native protein interactions, typically those involving mitochondrial membrane protein complexes 14. This separation technique was also recently used in combination with label-free protein quantification and correlation profiling, not only on mitochondrial complexes 15,16,17, but also for unravelling other protein complexes from whole cells 18,19. We hypothesized that the combination of affinity purification with subsequent native fractionation approaches and quantitative MS should provide a useful strategy for resolving multiple protein assemblies containing a particular protein.

Here we describe a method that combines generic epitope-based affinity purification with blue native polyacrylamide gel electrophoresis of the isolated complexes, followed by quantitative mass spectrometry and protein correlation profiling, to resolve the multiple assemblies a protein might be involved in. We employ mouse embryonic stem cells where a protein of interest fused to an epitope tag is expressed from the endogenous locus to achieve close to physiological abundance and ensure efficient native complex isolation. This approach unravels the multiple interactions a protein engages in, resolving them into distinct assemblies based on their correlation profiles, whilst requiring no more work than conventional geLC-MS 20.

## Protocol

### 1. Isolation of Native Protein Complexes by FLAG Affinity Purification

1. Preparations
  1. Set up a 37 °C water bath for thawing the cell pellet.
  2. Cool down a microcentrifuge to 4 °C.
  3. Place several microcentrifuge tubes of different sizes (1.5 mL, 15 mL) and a homogenizer in ice.
  4. Prepare 10 mL of lysis buffer (50 mM Tris-HCl pH 8, 150 mM NaCl, 0.1% Nonidet P-40, 1 mM EDTA) and keep in ice. Just prior to using the buffer, add 10 μL of 1 M DTT and a crushed tablet of EDTA-free protease inhibitors.
2. Preparation of antibody-linked beads NOTE: The antibody-coated beads can be prepared up to a week in advance and kept in the refrigerator until needed. Protein G has higher affinity than protein A for most species immunoglobulin subtypes, except for rabbit IgG, for which protein A has higher affinity.
  1. Wash 50 μL of Protein G-coated magnetic beads: Transfer 50 μL of bead slurry to a microcentrifuge tube, place the tube in the magnet to collect the beads on the side of the tube and remove the liquid. Resuspend the beads in 0.5 mL of PBS-0.01% Tween-20.
  2. Place the tube in the magnet to collect the beads on the side of the tube and remove the liquid. Resuspend the beads in 40 μL of PBS-0.01% Tween-20. Add 10 μg (10 μL of 1 mg/mL stock solution) of M2 anti-FLAG antibody. Incubate with rotation for 20 min at room temperature.
  3. Place the tube in the magnet to collect the beads on the side of the tube and remove the liquid. Wash the beads with 0.5 mL of PBS-0.01% Tween-20.
  4. For cross-linking the antibody to the beads, wash the beads twice with 1 mL of 0.2 M triethanolamine pH 8.2. Then, resuspend the beads in 1 mL of 20 mM DMP (dimethyl pimelimidate) in 0.2 M triethanolamine pH 8.2 (DMP solution should be prepared fresh immediately before use). Incubate with rotation at room temperature for 30 min.
  5. Place the tube in the magnet. Remove the supernatant and resuspend the beads in 0.5 mL of 50 mM Tris-HCl pH 7.5. Incubate with rotation at room temperature for 15 min.
  6. Wash the beads 3 times with 0.5 mL of PBS-0.1% Tween-20. Leave beads in the last wash until needed.
3. FLAG-based affinity purification NOTE: Take care in not leaving the beads without buffer for a long period of time. All buffers and tubes should be kept in ice at all times. The following protocol is optimized for the purification of protein complexes from 2-5 x 10^8^ cells (10-20 mg protein lysate) expressing endogenous levels of a FLAG-tagged protein 20.
  1. Thaw the cell pellet in a water bath at 37 °C until it starts to melt. Take the tube out of the bath, swirl it gently until the pellet is completely thawed, and immediately place the tube containing the cell suspension on ice.
  2. Add 5 mL of ice-cold lysis buffer (containing DTT and protease inhibitors) to the cell suspension and swirl to mix. Incubate in ice for 10 min.
  3. Transfer the lysate to a cold Dounce homogenizer. Lyse with 20-30 strokes using the tight pestle (until there is no noticeable viscosity). Transfer the homogenate to cold microcentrifuge tubes.
  4. Centrifuge at 18,000 x g for 15 min at 4 °C.
  5. Transfer the cleared lysate to a clean cold tube. Leave 50 μL of lysate behind. Take a 35 μL aliquot of lysate to measure protein concentration and monitor the purification.
  6. Remove the PBS-0.1% Tween-20 from the beads. Resuspend the beads with 1 mL of the lysate and mix with the rest of the lysate. Incubate the mixture in a rotating wheel at 4 °C for 1-2 h.
  7. Collect the beads on the side of the tube with the magnet. Collect a 30 μL aliquot of supernatant and discard the rest of the supernatant. Resuspend the beads in 0.5 mL of IPP150 buffer (10 mM Tris-HCl pH 8, 150 mM NaCl, 1mM EDTA, 0.1% NP-40) by pipetting 4-6 times. Transfer to a 1.5 mL cold tube.
  8. Repeat the washes with 0.5 mL of IPP150 buffer two more times.
  9. Wash beads three times with 0.5 mL of FLAG native elution buffer (20 mM Bis-Tris pH 7, 20 mM NaCl, 0.02% Nonidet P-40, 1 mM EDTA, 200 mM ε-aminocaproic acid). With the last wash, transfer the beads to a new cold tube. Remove buffer thoroughly.
  10. Resuspend the beads in 100 μL of 200 μg/mL 3x FLAG peptide in native elution buffer. Incubate at 4 °C for 10 min with gentle rotation.
  11. Collect the beads with the magnet and transfer the supernatant to a cold new tube.
  12. Repeat steps 1.3.10-1.3.11 twice. Pool all the eluates.
  13. Concentrate the eluate down to 25 μL in a centrifugal filter unit (10 kDa nominal molecular weight limit cut-off, PES) by centrifugation at 10,000 x g at 4 °C. Transfer the concentrated eluate to a new cold tube and add 50% glycerol for a final concentration of 5%. The concentrated eluate can be kept at 4 °C overnight if required.

### 2. Blue Native PAGE

1. Preparations
  1. Prepare 1 L of 1x native PAGE Anode Buffer, 1x native PAGE Dark Blue Cathode Buffer and 1x native PAGE Light Blue Cathode Buffer.
  2. Remove the comb of a precast 3-12% native PAGE gel and wash the wells twice with 1x native PAGE Dark Blue Cathode Buffer. Fill the wells with 1x native PAGE Dark Blue Cathode Buffer. Set up the gel in the running tank but do not add Dark Blue Cathode Buffer to the cathode (inside) chamber.
2. Electrophoretic run
  1. Prepare the sample: To the 25 μL concentrated eluate add appropriate volume of 4x native sample loading buffer and of 0.5% G-250 sample additive for final concentrations of 1x and 0.005% respectively.
  2. Load the sample and native molecular weight markers leaving an empty well between them. Load 10-20 μL of 1x native sample loading buffer in all the empty wells.
  3. Fill the cathode (inside) chamber with 200 mL of 1x native PAGE Dark Blue Cathode Buffer carefully so as not to disturb the samples. Fill the anode (outside) chamber with 550 mL of 1x native PAGE Anode Buffer.
  4. Run the gel at 150 V for 30 min. Stop the run, remove the Dark Blue Cathode Buffer with a serological pipette and replace with 1x Light Blue Cathode Buffer. Continue the run for 60 min. The gel can be run at room temperature or at 4 °C; the temperature might have an effect on protein complex structure.
3. Gel staining
  1. Open the gel cassette and discard one of the cassette plates. The gel remains attached to the other cassette plate. Transfer the gel into a tray or receptacle containing enough fixative solution (40% methanol, 2% acetic acid) to cover the gel and incubate for 30 min with gentle shaking.
  2. Remove the fixative solution and add enough water to cover the gel. The gel can be scanned for future reference.

### 3. Mass Spectrometry Analysis

Note: All subsequent steps should be performed in a laminar flow hood if possible to ensure cleanliness of the samples. All solutions should be prepared with HPLC-grade water. Discuss this protocol with the Mass Spectrometry laboratory that will carry out the analysis.

1. Preparations
  1. Place a conical bottom 96-well plate on top of another 96-well plate to collect the waste. Pierce the bottom of the wells of the conical bottom 96-well plate with a 21 G needle.
  2. Wash the wells of the pierced 96-well plate.
    1. Add 200 μL of 50% acetonitrile-0.25% formic acid to the wells of the pierced plate. Incubate the plate stack in a shaker for 10 min.
    2. Centrifuge at 500 x g for 1 min so that the liquid flows through the holes into the waste collection plate. Discard the waste liquid.
    3. Repeat steps 3.1.2.1-3.1.2.2 two more times.
    4. Fill the wells of the pierced 96-well plate with 150 μL of 50 mM ammonium bicarbonate (freshly made).
  3. Wash the wells of a centrifugal filtration 96-well plate.
    1. Place a normal 96-well plate under the centrifugal filtration plate to collect the waste. Add 200 μL of 50% acetonitrile-0.25% formic acid to each well of the centrifugal filtration plate.
    2. Centrifuge the plate stack at 200 x g for 1 min. Discard the waste.
    3. Repeat steps 3.1.3.1-3.1.3.2 two more times.
2. Gel excision and in-gel digestion NOTE: Whilst excising the gel, identify and make a note of the gel slice aligned to each of the protein standards. The protein standards can be used to estimate approximate molecular weights for all gel slices.
  1. Place the gel with the glass plate on a clean surface. Slice the lane containing the sample into 48 identical slices (1.5 mm x 5 mm). Cut each slice into 2-3 smaller pieces (except the last five slices at the top of the gel) and place each slice sequentially in one well of the pierced and washed 96-well plate.
  2. Add 50 μL of acetonitrile to each well. Incubate the plate with shaking for 30-60 min. Remove the liquid by centrifugation as in step 3.1.2.2.
  3. Add 200 μL of 2 mM TCEP in 50 mM ammonium bicarbonate to each well. Incubate the plate with shaking for 30 min at room temperature. Remove the liquid by centrifugation as in step 3.1.2.2.
  4. Add 200 μL of 4 mM iodoacetamide in 50 mM ammonium bicarbonate to each well. Incubate the plate with shaking for 30 min at 37 °C in the dark. Remove the liquid by centrifugation as in step 3.1.2.2.
  5. Add 150 μL of 50 mM ammonium bicarbonate plus 50 μL of acetonitrile and incubate the plate with shaking for 30 min at room temperature. Repeat this washing step as many times as required until all the blue color is removed from the gel pieces.
  6. Dehydrate the gel pieces by adding 200 μL of pure acetonitrile and incubate with shaking at room temperature for 20 min until the gel pieces are white and opaque. Remove acetonitrile.
  7. Add 150 μL of 0.001 mg/mL of trypsin (sequencing grade) in cold 50 mM ammonium bicarbonate to each well. Incubate the plate with shaking at 37 °C for 2 h. After 1 h, check that the gel pieces are covered with liquid; if this is not the case, add another 50-100 μL of 50 mM ammonium bicarbonate. After 2 h at 37 °C, continue the digestion at 25 °C overnight.
  8. Place a clean conical bottom plate under the gel-containing plate, ensuring correct orientation of the plates. Collect the liquid containing the peptides by centrifugation at 200 x g for 1 min.
  9. Place the peptides-containing plate in a centrifugal evaporator and evaporate the liquid whilst performing the next step.
  10. Add 150 μL of 50% acetonitrile-0.25% formic acid to the gel pieces. Incubate with shaking at 37 °C for 30 min.
  11. Collect the liquid into the partially dried plate from step 3.2.9 by centrifugation at 200 x g for 1 min. Continue evaporating the peptides-containing plate whilst performing the next step.
  12. Repeat steps 3.2.10-3.2.11.
  13. Add 200 μL of pure acetonitrile to each well of the plate containing the gel pieces. Incubate with shaking at room temperature for 15 min.
  14. Collect the liquid in the peptides-containing plate by centrifugation at 200 x g for 1 min.
  15. Make sure the final concentration of acetonitrile in all wells is above 55%. If required, add extra pure acetonitrile.
  16. Transfer the peptide solutions to the washed centrifugal filtration plate. Place a clean conical bottom plate underneath it to collect the peptides. Centrifuge the plate stack at 200 x g for 1 min.
  17. Evaporate the liquid in the conical bottom plate until wells are completely dry. The plate can be covered with a silicone lid and stored at -20 °C at this stage.
  18. Re-dissolve peptides in 32 μL of 0.5% formic acid with vigorous shaking for 15 min. Add 8 μL of 400 mM ammonium bicarbonate and incubate with vigorous shaking for 15 min. Centrifuge the plate at 200 x g for 1 min. Cover with a silicone lid and store the plate at -20 °C until mass spectrometry analysis.
3. Mass spectrometry NOTE: Analyze peptides on a high resolution mass spectrometer using methods compatible with shotgun proteomics. Data dependent acquisition is the most commonly used LC-tandem MS set-up for this type of analysis and should be optimized for efficient peptide identification, minimizing redundant sequencing and candidate selection over background noise. Mass spectra in the first stage mass analysis scan (MS1) should be recorded in profile mode. It is recommended to preferentially select doubly and triply charged ions for second stage mass analysis (MS2). A mass range of m/z 400-1,600 is suitable to identify most peptides between 8-30 amino acids range. Here, a protocol for analysis using an Orbitrap mass spectrometer is provided.
  1. Inject 20 μL of sample per analysis using the auto-sampler of a nanoLC-MS/MS system. Desalt and concentrate peptides on a reverse phase C18 column (100 μm id, 2 cm).
  2. Separate peptides on an analytical C18 column (75 μm id, 15 cm) using a 30 min linear gradient of 4-28% acetonitrile/0.1% formic acid at 300 nL/min.
  3. Analyze peptides in a mass spectrometer with a top 10 data dependent acquisition method with the following parameters: m/z range 380-1,600, resolution 30,000 at m/z 400, isolation width of 2 Th, dynamic exclusion at ± 10 ppm for 45 s, automatic gain control target at 1 x 10^6^ for MS and 5,000 for MS/MS, maximum injection time 150 ms for MS and 100 ms for MS/MS.

### 4. Data Analysis

1. Protein identification and quantification using MaxQuant
  1. Use the freely available MaxQuant software to identify and quantify proteins in each gel fraction from the mass spectrometry raw data. NOTE: MaxQuant works on data generated by data-dependent acquisition shotgun proteomics approaches. The MS data from the blue native gel fractions should be analyzed in batch as separate experiments and not as fractions of an experiment combining all gel slices. Check the iBAQ value box to perform stoichiometry calculations. Detailed protocols for database search and protein quantification using MaxQuant are described in 21,22.
2. Protein profile analysis using Perseus
  1. Use the freely available software Perseus to display the migration profiles of the identified proteins and compare them using statistical analysis. NOTE: General documentation on how to use Perseus is available at http://www.coxdocs.org/doku.php?id=perseus:user:tutorials. A sample dataset is available as supplemental data.
  2. Upload the proteingroups.txt file returned by the MaxQuant analysis containing the protein identifications and quantitation values for all gel fractions. Populate the Intensity values into the Main box (**Figure 1**). A matrix will appear containing all protein identifications in rows, with the gel fractions as columns.
  3. Remove entries corresponding to reverse hits, proteins only identified by site and potential contaminants by selecting Filter rows based on categorical column in the Filter rows dropdown menu (**Figure 2**).
  4. Normalize the fraction intensities of each protein across the profile against the total protein intensity by selecting Divide in the Normalize dropdown menu, then Sum (**Figure 3**). A new matrix appears.
  5. Display the migration profile plots of all proteins by selecting Profile Plot in the Visualization dropdown menu (**Figure 4**).
  6. Select Filter rows based on valid values in the Filter rows dropdown menu. Enter 1 in the number of valid values required.
  7. Perform hierarchical clustering of identified proteins across all blue native gel fractions by selecting Hierarchical clustering in the Clustering/PCA dropdown menu. Uncheck the Columns tree box (**Figure 5**). The clusters are visualized in a heat-map in the Clustering tab next to the matrix (**Figure 6**).
  8. Locate the cluster containing the bait protein in the dendrogram. Other components of the cluster represent proteins co-migrating with the bait and are therefore potential interacting proteins/subunits of the same complex.

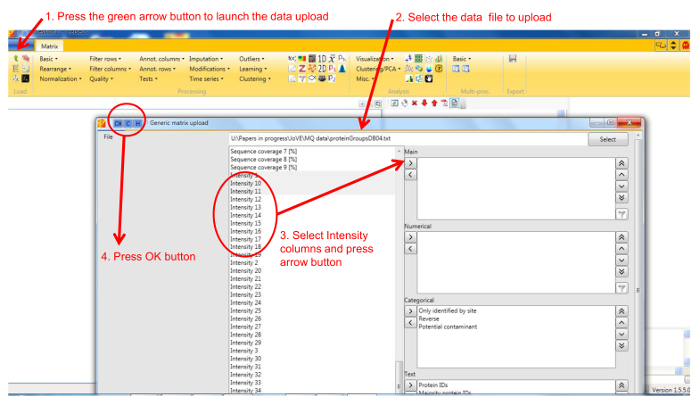
**Figure 1. Screenshot of Perseus data upload window.** The figure shows the steps for loading protein identification and quantification data into Perseus. Please click here to view a larger version of this figure.


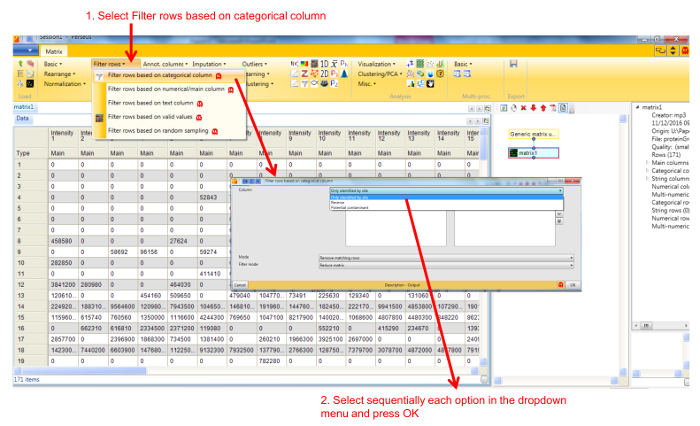
**Figure 2. Screenshot of Perseus window for filtering of entries corresponding to contaminants, reverse hits and proteins identified by site.** Selecting Filter rows by categorical column opens a new window for selecting the types of protein entries to be eliminated from the dataset. Please click here to view a larger version of this figure.


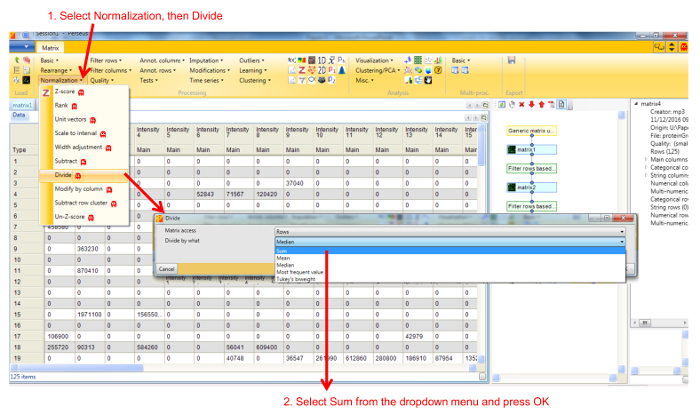
**Figure 3. Screenshot of Perseus normalization window.** Selecting Divide in the Normalization menu opens a new window, with different options. Selecting Sum divides the intensity value of a protein in each fraction by the total intensity of that protein across all fractions. Please click here to view a larger version of this figure.


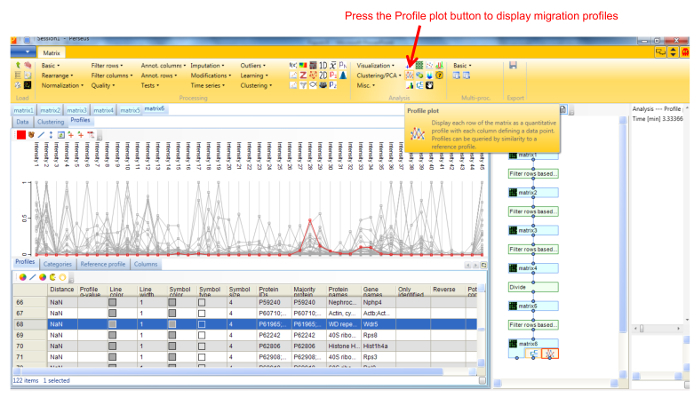
**Figure 4. Screenshot of Perseus showing migration profile plots.** Proteins can be selected in the matrix below the profiles to highlight their corresponding profile. The tool bar can be used to edit and export the profiles. Please click here to view a larger version of this figure.


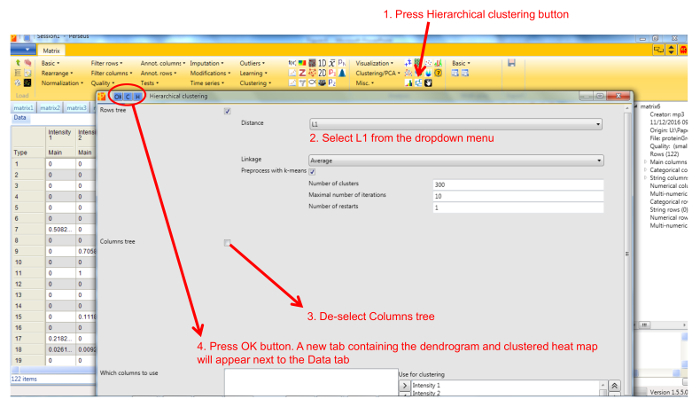
**Figure 5. Screenshot of Perseus hierarchical clustering window. **The figure shows the settings for hierarchical clustering of proteins using Manhattan (L1) distance metric. Please click here to view a larger version of this figure.


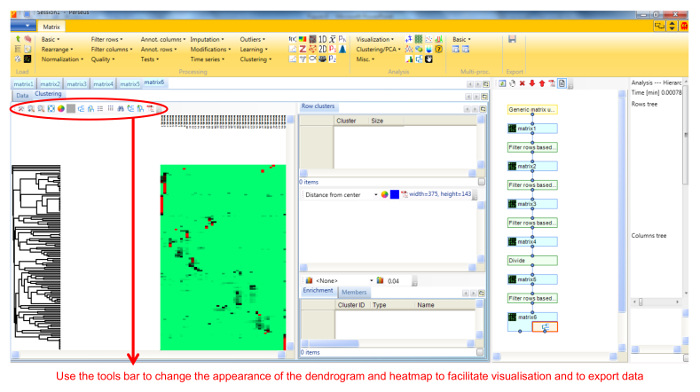
**Figure 6. Screenshot of Perseus showing dendrogram and protein intensities heatmap. **The workflow on the right hand side of the main panel shows each step undertaken and the resulting matrix, and can be used to undo any step. The workflow can also branch from any intermediate matrix. Please click here to view a larger version of this figure.

1. Calculation of relative stoichiometry
  1. Select the subunits of a protein complex based on their co-clustering with the bait protein and delimit the profile peak(s) where they appear.
  2. Use the iBAQ values returned by MaxQuant analysis for each subunit in each fraction separately and normalize by the corresponding bait iBAQ value in that fraction.
  3. Plot the normalized iBAQs for bait and protein complex subunits across the fractions of the selected peak and apply linear regression. Calculate relative amount of complex subunit to bait by comparing the trends of their normalized iBAQ values.
2. Estimation of protein complex size
  1. Use the protein standards to calculate predicted molecular weights for the aligning blue native gel slices from the sample lane. From these, generate a linear regression model by plotting gel slices in the *x*-axis and molecular weights in the *y*-axis.
  2. Use the model to estimate the observed molecular weight range of the protein complex(es) based on the blue native slice(s) from which they were identified.

## Representative Results

The workflow of the Affinity purification Blue native protein Correlation profiling by Mass Spectrometry (ABC-MS) strategy is depicted in **Figure 7**. Native protein complexes around a protein of interest are isolated by affinity purification using antibodies against an epitope tag (in this case FLAG) and competitive elution. The complexes are resolved by BN-PAGE, and the whole gel lane is excised into 48 sections, and prepared for shotgun LC-MS/MS. Quantitative MS information is used to generate a migration profile for each identified protein across the blue native separation. Proteins that interact to form a distinct complex display similar migration profiles with superimposable peaks. When a protein of interest takes part in more than one assembly, multiple peaks are observed in its migration profile, given the sub-complexes are within the resolving power of the blue native gel. A systematic comparison of the migration profiles can be achieved by protein correlation profiling using hierarchical clustering. The dendrogram and the peak intensities for all fractions are visualized in a heat-map, facilitating the identification of interacting proteins that belong to distinct protein complexes (**Figure 8**).

We used this strategy to analyze the interacting partners of Mta2, a core subunit of the NuRD chromatin remodeling complex 20. As shown in **Figure 9**, the migration profile of Mta2 displayed two peaks of distinct intensities between 700 kDa and 1.2 MDa, with the lower mass peak displaying higher abundance. Other NuRD core subunits, including Mta1/3, Hdac1/2 and Mbd3, showed identical separation pattern, albeit the peaks for some of the subunits, namely Chd4, Gatad2a/b and Rbbp4/7, had the inverse abundance distribution (**Figure 9A, B**). In contrast, the profile of Cdk2ap1, a regulatory factor that recruits the NuRD complex to Wnt gene promoters 23, only displayed the higher mass peak (**Figure 9C**), and so did Sall4, a transcriptional repressor that has also been shown to bind NuRD 20,24,25. Thus, fractionation of affinity purified Mta2-associated proteins by blue native PAGE was able to resolve two different forms of the NuRD complex.

ABC-MS also allows the identification of novel interactors whilst assigning them to particular protein entities. Through examination of the proteins clusters and fraction intensities represented in a heat-map we detected a strong correlation between NuRD subunits and Wdr5, a regulatory subunit of the MLL methyltransferase complex 26, with Wdr5 displaying two migration peaks coincident with the two NuRD peaks (**Figure 8**), suggesting a novel interaction between Wdr5 and NuRD. We confirmed this interaction by co-immunoprecipitation and co-migration in size exclusion chromatography 20.

The choice of distance metric calculation in the hierarchical clustering will influence the shape of the clusters and hence the correlations. We recommend experimenting with the different metrics to achieve the best fit with existing interaction knowledge of the protein or complex of interest. Generally we achieved best results with the Manhattan (L1) distance metric 20. Pearson correlation or Euclidean distance metrics have also been reported for identifying complexes based on alternative fractionation techniques 10,11,12.

The quantitative MS data obtained from the blue native fractions can also be used to determine the stoichiometry of protein complexes. The iBAQ (intensity based absolute quantification) value provides a measure of relative abundance of the identified proteins 27,28. The iBAQ values for the protein complex members in all the fractions within a migration profile peak are normalized to that of the bait protein to obtain relative amounts. The normalized iBAQs across a profile peak for a given protein complex member should follow a horizontal trend, and the trend values reflect the stoichiometry of the interacting proteins relative to the bait protein. For a more detailed representation of stoichiometry calculations, see 20.


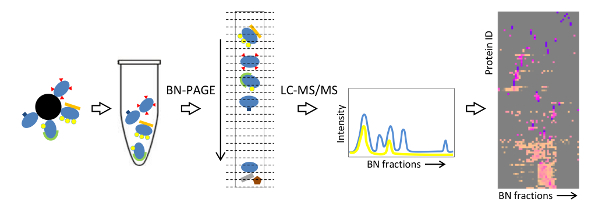
**Figure 7: Schematic workflow summarizing the ABC-MS strategy.** This figure is modified from 20. Please click here to view a larger version of this figure.


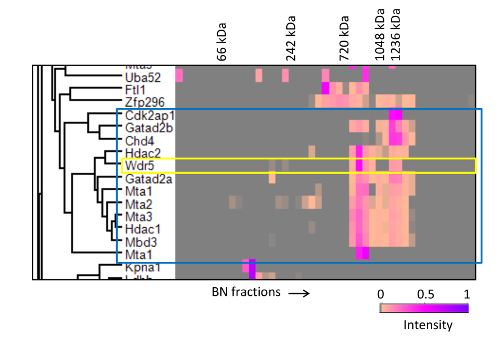
**Figure 8. Hierarchical clustering of BN-PAGE migration profiles of Mta2 interactors.** Mta2-associated proteins were clustered based on the similarity of their migration profiles. Only a subset of the heat-map containing the NuRD complex is shown (enclosed in the blue box). The yellow box highlights the strong correlation of Wdr5 with the NuRD complex. The annotated molecular weights were estimated from the migration distances of protein standards run in the same gel. This research was originally published in Molecular and Cellular Proteomics 20 the American Society for Biochemistry and Molecular Biology. Please click here to view a larger version of this figure.


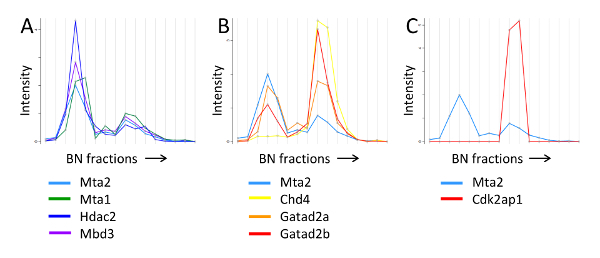
**Figure 9. BN-PAGE migration profile of NuRD subunits and associated proteins. A)** Mta2 and NuRD subunits present at a similar intensity pattern. **B) **Mta2 and NuRD subunits with the inverse intensity pattern. **C) **Mta2 and Cdk2ap1, which is present only in the higher molecular weight NuRD entity. The annotated molecular weights were estimated from the migration profile of the protein standards. This research was originally published in Molecular and Cellular Proteomics 20 the American Society for Biochemistry and Molecular Biology. Please click here to view a larger version of this figure.


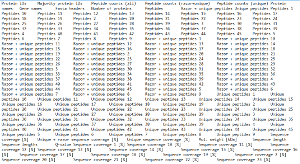
**Supplemental data: Sample dataset.** MaxQuant-derived proteingroups.txt file containing protein identifications and quantification values from an ABC-MS experiment. This research was originally published in Molecular and Cellular Proteomics 20 the American Society for Biochemistry and Molecular Biology. Please click here to download this file.

## Discussion

Here, we describe the use of affinity purification followed by blue native gel electrophoresis in combination with quantitative mass spectrometry to resolve protein complexes. This approach offers a method to unravel one-dimensional protein interaction lists into functional protein assemblies.

We demonstrate the method based on the use of epitope-tagged proteins. However, if a cell line expressing a tagged protein is not available, an alternative might be to use antibodies against the protein of interest, provided there is a peptide available to achieve native competitive elution. The amount of starting material and quantity of beads might need to be modified depending on the expression level of the target protein. We typically perform this protocol with 2-5 x 10^8^ cells starting material, and this is sufficient even for proteins with low expression level. The lysis buffer composition should be chosen empirically to achieve near to complete solubilization of the bait. This might be challenging for some types of proteins, in particular chromatin binding or membrane proteins. Alternative options include increasing the amount of salt, provided that the protein complex under study is stable in high salt, or using sonication and/or nuclease treatment for chromatin binding proteins 20,29. In the case of membrane proteins, switching detergent to DDM or digitonin may be advisable 30. In the case of DNA binding proteins, it is useful to include a nuclease such as benzonase during the purification step 20. Complete removal of nucleic acids ensures that the interactions detected occur between proteins and are not mediated by DNA.

A critical element to consider when using this approach is the stability of the complex under investigation. The procedure is long and may involve preserving the protein complex overnight. We have achieved good success with two chromatin remodeling complexes (D. Bode and M. Pardo, data not shown), but this should be evaluated. The affinity purification step may be shortened if required.

Alternative techniques that allow native fractionation, such as size exclusion chromatography, have been widely used for over 50 years to characterize protein complexes. We and others have shown that the resolution of blue native PAGE is superior to that achieved using size exclusion chromatography 20,31,32,33. Another advantage of blue native PAGE is that it does not require chromatography systems, which are expensive, but rather uses protein electrophoretic equipment that is widespread in laboratories. In terms of hands-on time, this method does not involve more work than the traditional geLC-MS/MS approach or offline chromatographic fractionation. However, as most fractionation techniques do, it has a limit to their resolution. Complexes that are very homogeneous or close in mass and shape may be beyond the resolution offered by blue native PAGE, and hence the protocol as reported here might not be universally successful in resolving distinct complexes with shared subunits. Encouragingly, we have been successful in separating two very similar tetrameric complexes sharing three subunits (M. Pardo, manuscript in preparation).

Since mass spectrometry has become increasingly sensitive, even minute amounts of non-specific interactors and contaminants can be detected in AP-MS samples. The approach presented here might aid in the discrimination of real interactors from background contamination in the affinity purification by focusing the attention on proteins with migration peaks that coincide with bait migration peaks at higher molecular weight than that of the monomeric proteins.

Several groups have used fractionation techniques followed by protein correlation profiling to delineate protein complexes at cellular scale without the need for previous isolation 10,11,12,34. However, this can result in failure to detect sub-stoichiometric interactions. The incorporation of an enrichment step through affinity purification can help to overcome this. The approach described here should be generally useful for exploring the topology of protein complexes and unraveling the multiple complexes a given protein takes part in within the same cellular context. The strategy is simple and amenable to laboratories that may not have expensive chromatographic fractionation equipment to resolve protein complexes.

## Disclosures

The authors have nothing to disclose.
